# The use of social media for professional purposes among dentists in Saudi Arabia

**DOI:** 10.1186/s12903-021-01390-w

**Published:** 2021-01-12

**Authors:** Khalifa S. Al-Khalifa, Abdullah S. Al-Swuailem, Rasha AlSheikh, Yasmeen Y. Muazen, Yazeed A. Al-Khunein, Hassan Halawany, Khalid S. Al-Abidi

**Affiliations:** 1 Present Address: Department of Preventive Dental Sciences, College of Dentistry, Imam Abdulrahman Bin Faisal University, P.O. Box 1982, Dammam, 31441 Saudi Arabia; 2 Department of Periodontics and Community Dentistry, College of Dentistry, King Saud University, Riyadh, Saudi Arabia; 3 Department of Restorative Dental Sciences, College of Dentistry, Imam Abdulrahman Bin Faisal University, Dammam, Saudi Arabia; 4 Dental Internship Program, College of Dentistry, Imam Abdulrahman Bin Faisal University, Dammam, Saudi Arabia; 5 Dental Department, Armed Forces Hospital, Dhahran, Saudi Arabia; 6 Department of Substitutive Dental Sciences, College of Dentistry, Imam Abdulrahman Bin Faisal University, Dammam, Saudi Arabia

**Keywords:** Social media, Opinion, Dentists, Healthcare, Saudi Arabia

## Abstract

**Aim:**

To investigate the dentists’ opinions towards social media (SM) use in daily practice and the expected limitations from its use in Saudi Arabia.

**Methods:**

An electronic survey was carried out throughout May–June 2020 among a sample of dentists in Saudi Arabia. The survey covered three parts: the first part covered professional and demographic information, the second part covered the use of mobile phones and SM in dental practice, while the third part assessed dentists’ opinion on SM use. Descriptive statistics included frequency distributions and percentages and independent *t *test/ANOVA test for the relationship between the mean of dentists’ opinion towards SM and demographic variables. A p value of 0.05 or less was considered statistically significant.

**Results:**

The majority of respondents (80%) believe that SM plays an active role in patients’ decisions regarding the selection of a healthcare provider. The mean dentists’ opinion scores on the use of SM were significantly lower among participants working more than 50 h per week compared with other participants (p = 0.014).

**Conclusion:**

The majority of sampled dentists believe that SM plays an active role in patients’ decisions regarding the healthcare provider’s selection. Directed campaigns can help dentists optimize the use of SM for both professional and personal purposes.

## Introduction

There is an increase in social media (SM) use due to the increase in technological advancement. It has changed how individuals communicate and share information. People nowadays are more dependent on SM to explore available services, including dental services viewing displayed information, customers’ feedback, and reviews. Hence, rendering visible communication as an essential part of any dental clinic activity [1, 2]. Dental providers’ SM’s engagement is growing every day, becoming a tool that helps them connect, learn, involve professionally, and assist in dental care [3, 5]. Proper communication with patients is one of the primary factors of success for any healthcare provider [6]. The SM platforms also have proven to be multi-faceted, offering a wide variety of tools, such as interactive blogs and audio-visual dissemination arenas catering to a broad audience who are potential future patients [3]. The micro-blogging site Twitter has also gained popularity among the medical fraternity to disseminate medical knowledge [5]. Out of the 168 Twitter accounts reported by Sugawara et al. [4], 73 were related to dentistry and oral surgery. SM has proven to be an effective and easy method for educating the laypeople and general masses [7].

There have been mixed reviews on the benefits of using SM by healthcare providers. The most-reported concerns were legal and security issues [8–11]. Counts of reviews of the medical literature available online have been labeled “low quality” which, if fallen into the wrong hands and taken heed of, could lead to potentially adverse, possibly lethal consequences such as drug overdose or unnecessary cosmetic surgical procedures [5]. In addition, SM tends to spread misinformation much quicker than reliable and verifiable facts, which might cause cyber disarray or confusion. This could lead to breaches in patient-healthcare provider confidentiality, professional image ruin, and healthcare professionals licensing issues [10]. Not to mention the amount of distortion a piece of information can go through being forwarded from one SM platform to another amongst laypeople [7].

Nevertheless, SM could improve the healthcare provision, especially with marketing, education, communication, and patients’ condition follow-up [10–12]. SM is increasingly being used as a marketing scheme for organizational visibility. This increases the chances of channeling patients towards organizations that post ads on various SM platforms boasting about better customer support and efficient service provision [10]. Increased SM use in Europe for health communication has been observed, with around 22% of Norwegian hospitals using the SM platform Facebook for health communication [13]. Better visibility and interactions with potential patients imprinting a positive image on their minds lead to a better business sustainability at virtually reduced costs [12].

Several studies explored the perception of dental and other healthcare providers towards the use of SM [14]. More than half of dental practitioners surveyed in one study believed that SM platforms are more effective in marketing than conventional methods [11]. Parmar et al. [6] revealed a positive attitude toward SM’s use to attract new patients. In a Saudi Arabian Study, one-third of the participants mentioned that they use SM to communicate with their patients and market their practice [14]. In another study that targeted physicians in Saudi Arabia, most of the participants stated that SM had a good impact on physicians’ knowledge and abilities; however, there were ethical concerns regarding its use [3]. Ranschaert et al. [15] highlighted the need to create a clear guideline to improve physicians’ skills in using SM safely and professionally. At the same time, another study specified that SM’s role in the dental-care provision is still a vague area for both patients and dentists. Both share concerns about its uses and benefits and noted the excellent opportunity for dental practices to utilize and benefit from the use of SM [6]. Given limited studies covering the use of SM by dentists in Saudi Arabia, this study aims to investigate dentists’ opinions towards the use of SM in daily practice and the expected limitations from its use.

## Materials and methods

The present cross-sectional study was conducted between May and June of 2020 on a sample of dentists in Saudi Arabia. Study subjects were invited to participate in this study voluntarily. A convenience sample was selected from SaudiDent.com database, which contains approximately 5000 dentists in Saudi Arabia. The sample size was calculated based on a 95% confidence level, a 5% margin of error, and a 50% response distribution. The minimum required sample size was determined to be 357 (http://www.raosoft.com/samplesize.html). To accommodate for non-responders, the sample size was increased by 10% (i.e.: n = 393).

The questionnaire was adapted from previously validated questionnaires used in similar studies on the use of SM targeting medical professionals [3, 16]. The survey questionnaire contained 16 questions. The first part of the questionnaire included professional and demographic information such as age, gender, qualification, work experience in years, region, work setting, and working hours per week. In the second part, it included the use of mobile phones and SM in dental practice such as daily general-purpose use of SM (in hours), preferred communication tool in dental practice with patients, the frequency of using any of the SM platforms and the type of SM platform provided by the employers. In the third part, it explored dentists’ opinion on SM use such as discussing internet or SM usage with their patients (Yes, No, Unsure), role of SM in improving their professional knowledge and skills (Yes, No, Unsure), dentist’s responsibility to disprove inaccurate health information posted online (Yes, No, Unsure), appropriateness of searching for patients’ personal information on SM as part of regular clinical practice (Disagree, Neutral, Agree), patients’ confidence of professional advice obtained by treating dentist from mobile phone applications or websites (Disagree, Neutral, Agree), preference of conducting a consultation with a patient via skype (or other online telecommunications) (Yes, No, Unsure), and their beliefs on whether SM would affect the patients’ selection of healthcare provider (Disagree, Neutral, Agree).

The survey was pretested on a pilot group of 20 general dentists [a reliability coefficient (alpha) of 0.75] before distribution to ensure questions clarity and overall acceptability of the survey. Minimal corrections were made based on the feedback obtained from the pilot group subjects. Since this was a questionnaire-based study, an exemption was granted for this study by the Ethical Committee of the College of Dentistry, Imam Abdulrahman Bin Faisal University. An informed consent was obtained from all subjects. In addition, this study was carried out in accordance with relevant guidelines and regulations.

The survey was created using SoGoSurveys® software [17]. It was then distributed to selected subjects via WhatsApp, Facebook, Twitter, and Instagram. A reminder message was sent on a weekly basis as means of follow-up for non-respondent practitioners.

The data was entered in MS Excel (2010) and transferred to IBM SPSS Statistics for Windows, version 22 (IBM Corp., Armonk, NY, USA) for statistical analysis. Descriptive statistics included frequency distributions and percentages. Mean of dentists’ opinion (Main outcome) was calculated and used for bivariate analyses purposes. The significance between the mean of dentists’ opinion on the use of SM and demographic variables was tested using an independent *t *test for dichotomized independent variables and ANOVA test for the other independent variables. A p value of 0.05 or less was considered statistically significant.

## Results

Between May and June 2020, 1000 surveys were sent out to dental practitioners, 392 responses were returned, indicating a response rate of 39.2%. Out of 392 participants, 364 participants responded with the completed survey (survey completion rate of 92.8%). The demographic information of the 364 study participants is shown in Table 1. Most of the participants (58.5%) were males, more than half of the participants (62.9%) were less than 35 years old, and about one third of them (38.2%) belonged to the central region of Saudi Arabia. In addition, 40.7% of the participants were general dental practitioners, and 26.4% were consultants/specialists. Similarly, half of the surveyed dentists have less than five years of experience. Most of the participants work in a governmental job, and a majority of them (94.2%) work for less than 50 h per week. About half of the participants (48.4%) spent less than 3 h per day in daily general-purpose use of SM. While 42.6% of the participants prefer phones as a communication tool in their dental practice with patients.

**Table 1:** Demographic information of study participants (n = 364)

Variables	N (%)
*Age in years*	
< 35 years	229 (62.9)
≥ 35 years	135 (37.1)
*Gender*	
Male	213 (58.5)
Female	151 (41.5)
*Qualification*	
Consultant/specialist	96 (26.4)
General dental practitioner	148 (40.7)
Resident/graduate research	59 (16.2)
Dental intern	61 (16.8)
*Work experience in years*	
0–5 years	183 (50.3)
6–10 years	88 (24.2)
11–15 years	30 (8.2)
> 16 years	63 (17.3)
*Region of the main job*	
Eastern	125 (34.3)
Central	139 (38.2)
Southern	18 (4.9)
Western	63 (17.3)
Northern	19 (5.2)
*Work setting of the main job*	
Private	67 (18.4)
Governmental	209 (57.4)
Both (private and governmental)	33 (9.1)
Academic	55 (15.1)
*Working hours per week*	
1–19 h	68 (18.7)
20–34 h	84 (23.1)
35–49 h	191 (52.5)
50 + hr	21 (5.8)
*Daily general-purpose use of social media (in h)*	
< 30 min	21 (5.8)
> 30 min to < 3 h	155 (42.6)
> 3 to < 6 h	131 (36)
> 6 h	57 (15.7)
*Preferred communication tool in your dental practice with patients*	
Social media	95 (26.1)
E-mail	21 (5.8)
Phone	155 (42.6)
In-person	93 (25.5)

**Fig. 1:**
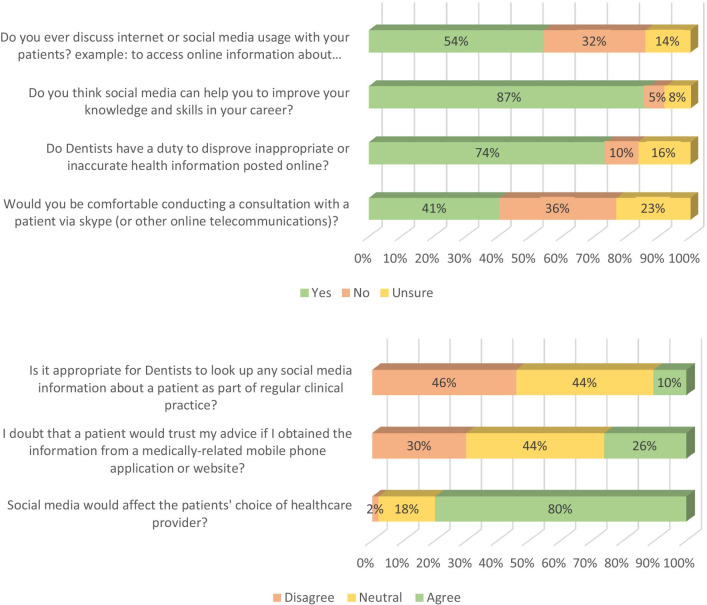
Dentists’ opinion scores on the use of social media

Dentists’ opinion of the use of SM in their practice is presented in Fig. 1. More than half of dentists (54%) encourage their patients to search the internet or SM to access online information about their condition. When asked if SM can help improve dentists’ knowledge and skills, 87% of the respondents confirmed it. Regarding inaccurate health information in SM, most sampled dentists (74%) believed that they have professional obligations to correct any incorrect information. While only 41% of the surveyed dentists were willing to conduct consultations online, 36% preferred conventional communication with patients.

Only 10% of sampled dentists considered using SM as a tool to collect personal information about their patients as appropriate. About 26% of the dentists agreed that their patients would doubt their clinical advice if they use a medically related mobile phone application or website. The majority of sampled dentists (80%) believe that SM plays an active role in patients’ decisions to select healthcare providers.

Table 2 shows the relationship between the respondents’ age with the daily use of SM. Younger participants mostly used Twitter, WhatsApp, Instagram, Youtube, and Snapchat platforms compared to older ones. Statistically, a significant difference was observed in proportions of daily Twitter, Facebook, Instagram, and Snapchat use between younger and older participants with p value < 0.05.

**Table 2:** The association between respondents’ age and the type of social media usage

Type of social media	Age group in years		p value*
	< 35 years	≥ 35 years	
	n (%)	n (%)	
Twitter	154 (67.2)	67 (49.6)	0.001*
WhatsApp	221 (96.5)	128 (94.8)	0.3
Facebook	17 (7.4)	32 (23.7)	0.001*
Instagram	140 (61.1)	52 (38.5)	0.001*
YouTube	132 (57.6)	66 (48.9)	0.065
LinkedIn	9 (3.9)	5 (3.7)	0.577
Snapchat	187 (81.7)	50 (37)	0.001*

*Significant at p = 0.05

Table 3 shows a comparison of mean dentists’ opinion scores on the use of SM among different demographic factors. The mean dentists’ opinion scores on the use of SM were significantly lower among participants working more than 50 h per week compared with other participants (p = 0.014).

**Table 3:** Comparison of demographics and mean dentists’ opinion scores on the use of social media

Variables	Mean ± SD^a^	p value
*Age in years*		
< 35 years	12.5 ± 1.8	0.721
≥ 35 years	12.4 ± 1.9	
*Gender*		0.638
Male	12.4 ± 1.9	
Female	12.5 ± 1.6	
*Qualification*		0.613
Consultant/specialist	12.3 ± 1.9	
General dental practitioner	12.5 ± 1.9	
Resident/graduate research	12.5 ± 1.7	
Dental intern	12.7 ± 1.7	
*Work setting of the main job*		
Private	12.6 ± 1.8	0.410
Governmental	12.4 ± 1.8	
Both (private and governmental)	11.9 ± 1.6	
Academic	12.6 ± 1.8	
*Working hours per week*		
1–19 h	12.6 ± 1.6	
20–34 h	12.4 ± 1.9	0.014*
35–49 h	12.5 ± 1.7	
50 + h	11.6 ± 1.8	

*Significant at p = 0.05

^a^Possible maximum opinion score = 17

## Discussion

This study’s findings agree with the results of several reports that younger-aged dentists are using SM to engage with their patients compared to older-age dentists [18, 19]. When considering a dentist’s age as a determinant factor in using SM, younger dentists (under 35 years old) were using Twitter, Instagram, and Snapchat significantly more than older dentists. It is worth to mention that social media is relatively recent, where social platforms such as Instagram and Snapchat started only 10 years ago while Twitter started 14 years ago. Thus, someone could realize the larger effect of social media on younger generations who grew up with social media surrounding them.

Dentists must understand that SM’s professional use should be dictated by the type of SM frequently used by their patients. Furthermore, the daily use of SM was alarming as most respondents reported using SM for more than 30 min a day, which might introduce signs of SM “over-dependence” [20]. This reported overuse of SM needs to be addressed by dentists and professional organizations in the form of educational programs and counseling services to better guide dental professionals in the proper use of SM.

The effect of SM on dental care delivery is undisputable. Patients use SM to collect information on their health status, health concerns, and health care providers [21–23]. Our study’s findings confirmed the effect of SM, as the majority of sampled dentists believed that a high proportion of patients are using SM to choose their treating dentists. Ajwa et al. reported that 89.4% of dental practitioners believed that SM is the most effective marketing strategy to recruit patients into dental practices in Saudi Arabia. They also reported that 82.3% of their sampled participants mentioned that posting an ad on SM created an increased influx of patients to the dental clinics [14]. This is in line with the psyche of the current generation as they like to explore their options on SM before they embark on the journey, be it their doctor’s appointment or their travel expenses. It gives a sense of security because they back their information obtained on SM and the internet.

Because of the importance of SM’s role in shaping the dental practice, it is not surprising that more than half of the sampled dentists in this study reported discussing SM usage with their patients. Concerning the accuracy of health information on SM, Sumayyia et al. said that among other issues, addressing information accuracy may reduce the risk of misleading information to the patients [24]. This contribution could be made possible by encouraging patients and general masses to access repute websites with scientific rigor and informing their patients in the process of how to differentiate between websites of good and bad scientific quality.

In our study, most sampled dentists (74%) believe that dentists should take a leading role in rectifying inaccurate online health information. For this reason, Koumpouros et al. suggested that SM should be useful in marketing, gaining patients’ trust and covering their needs [25]. As also put forth by Mangold et al., the relationship between the originators of a healthcare message and the laypeople who read that message is changing and evolving constantly. Hence, a certain degree of control is required for healthcare professionals using SM platforms to manage the content validity and reliability reaching the laypersons through the internet, as misinformation is rampant and could have fatal consequences [26]. Bahkali et al. reported the importance of the accuracy of the health information available online. It can be used as a strengthening means to improve the health care system [27], and 74% of this study participants believe that dentists need to disapprove and clarify inappropriate or inaccurate online health information. This reflects an understanding of the situation about patients’ needs and in agreement with published literature.

No doubt that SM has made a significant change in the health profession in recent years. Part of this change is related to knowledge gain and improved clinical judgment. In our study, 86% of sampled dentists believed that SM could improve their knowledge and skills and promote their careers. These findings agree with similar literature [28, 29]. The ease of contact between healthcare providers and the public is one of SM’s strengths. Parmar et al. reported that about 44% of sampled patients liked the idea of being in contact with their dentists via SM [6]. In addition, Henry et al. reported that 52% of dentists contact their patients on Facebook [30]. In our study, less than half of the study sample were willing to provide dental consultation through SM. It is possible that the unwillingness of the majority of sampled dentists to provide dental consultation on SM could be related to inadequate information to make such consultation or fear of legal consequences for such consultation.

One of SM’s critical issues is related to ethical and privacy perspective [30, 31]. In this study, less than half of the respondents believed it is inappropriate for dentists to check their patient’s SM account. Lack of engagement on SM because of privacy issues has been previously reported [32]. Only 41% of the study sample felt comfortable conducting a consultation with patients. Clear-cut boundaries between medical professionalism and SM indiscretion need to be defined beforehand because new medical and dental students being inducted in respective programs already have a sense of technology applications being used to share information, leaving what is now being called a “digital footprint” for others to see [33]. Although SM usage has to be encouraged; some boundaries and guidelines are needed; misuse represents a significant risk to the individuals using or in charge of monitoring SM use in the clinical practice. Clear policies, limitations, and aspects of service should be available for dental personnel to reduce the risks [34–37].

Although close to a reported percentage by the published literature, this study’s results still reflect some conflict; participants are shy to be engaged themselves. Up to 70% of the participants doubted that patients would trust advice or information provided online or by phone. Together with the dentists, patients should develop critical appraisal skills to apply to the information posted on SM and be able to judge which is appropriate and trustworthy [38]. Targeted educational programs should be established to help dentists utilize SM, conduct a virtual clinic or learning sessions that might be advantageous, and be designed to target practicing dentists or undergraduate students [3].

The use of SM depends on several demographic factors, among which age plays an essential factor. To our surprise, there was no difference between older and younger participants favoring SM’s use. One study on US dental educators reported that older dental educators favored SM use [32]. On the other hand, two studies reported that younger dentists favored SM more than older dentists [30, 39]. A possible explanation for having older participants in our study favor SM’s use would be that most of them are consultants and specialists who have been trained in the US and Europe and have private practices. Most of them have SM accounts focused on their clinical practice. In our study, gender and qualifications were not contributing factors in using SM, especially when using SM for business purposes. Snyman and Visser made a similar observation among their sample of South African dentists. However, when SM is used for personal purposes, female dentists tend to favor using SM more than male dentists [40].

In this study, working experience had no effect on the use of SM. This observation is not similar to some other reported studies. For example, one study conducted in Ecuador reported that dentists with more than eight years of experience were associated with a lower likelihood of using SM [39]. Furthermore, it has been reported that there was no association between years of experience and SM use among sampled dentists in one study in South Africa [40]. This difference in SM use among experienced and less-experienced dentists could be explained in light of differences in people’s behaviors and attitudes toward SM among different nations and cultures.

Interestingly, the type of job setting did not affect SM’s use for the participants in this study. However, the number of working hours per week showed an association with the dentists’ opinion towards the use of SM. Those who worked less than 20 h per week scored higher than those who worked more than 50 h per week. This could be explained by the fact that consultants might be working fewer hours than general dentists, thus coinciding with the scores for both age and work experience in years where older and more experienced dentists scored higher for SM use.

This study sheds light on SM’s importance in dental practice as more dentists and patients are reliant on this form of technology. Dental practice can be enhanced by the SM’s use in the provision of dental services, advertising, counseling and oral health education. SM platforms could also be used for professional development, where dental organizations and dental educators can disseminate information and updates via different SM platforms. Nevertheless, future studies to examine the impact of individual SM platforms on dental practice and dental education is needed.

## Limitations

This study has some limitations. One of these limitations is related to the method used to collect relevant information through an electronic survey. There is the good proportion of the targeted population that either does not respond to electronic surveys or does not use such a communication method. Second possible limitation related to the sample being mainly from central and eastern regions (72%). The western region is second to the central region in both the number of Saudi population living in this geographic location and the number of practicing dentists. Thirdly, it is quite challenging to discern between active users of SM and users of SM by word of mouth. The fourth possible limitation is the lack of a probability sampling technique. This may affect the generalizability of results to the whole dentist population in Saudi Arabia. Also, fewer survey participation from dentists in the private sector. This could be explained by the fact the Saudi dentists generally favor working in governmental sector.

## Conclusion

The majority of sampled dentists believe that SM plays an active role in patients’ decisions regarding the healthcare provider’s choice. SM is essential for the success of patients’ engagement and practice marketing. Taking this belief into consideration, directed campaigns can help dentists optimize the use of SM to their benefit without compromising integrity; such a campaign can help those who are shy to be engaged or those who still have concerns. Despite this study’s limitations, it can help shed light on areas that require further investigation and exploration, such as limitations, over-dependence, and confidentiality.

## Data Availability

The data supporting the findings of this study are available upon reasonable request from the corresponding author.

## Abbreviation

SM: Social media
